# Novel antimicrobial agents targeting the *Streptococcus mutans* biofilms discovery through computer technology

**DOI:** 10.3389/fcimb.2022.1065235

**Published:** 2022-12-01

**Authors:** Bin Zhang, Min Zhao, Jiangang Tian, Lei Lei, Ruizhe Huang

**Affiliations:** ^1^ Key Laboratory of Shaanxi Province for Craniofacial Precision Medicine Research, College of Stomatology, Xi’an Jiaotong University, Xi’an, China; ^2^ Clinical Research Center of Shaanxi Province for Dental and Maxillofacial Diseases, Center of Oral Public Health, College of Stomatology, Xi’an Jiaotong University, Xi’an, China; ^3^ State Key Laboratory of Oral Diseases, Department of Preventive Dentistry, West China Hospital of Stomatology, Sichuan University, Chengdu, China

**Keywords:** extracellular polymeric substances, drug repurposing, computer-aided drug design, synthetic antimicrobial peptides, computer technology

## Abstract

Dental caries is one of the most prevalent and costly biofilm-associated infectious diseases worldwide. *Streptococcus mutans* (*S. mutans*) is well recognized as the major causative factor of dental caries due to its acidogenicity, aciduricity and extracellular polymeric substances (EPSs) synthesis ability. The EPSs have been considered as a virulent factor of cariogenic biofilm, which enhance biofilms resistance to antimicrobial agents and virulence compared with planktonic bacterial cells. The traditional anti-caries therapies, such as chlorhexidine and antibiotics are characterized by side-effects and drug resistance. With the development of computer technology, several novel approaches are being used to synthesize or discover antimicrobial agents. In this mini review, we summarized the novel antimicrobial agents targeting the *S. mutans* biofilms discovery through computer technology. Drug repurposing of small molecules expands the original medical indications and lowers drug development costs and risks. The computer-aided drug design (CADD) has been used for identifying compounds with optimal interactions with the target *via* silico screening and computational methods. The synthetic antimicrobial peptides (AMPs) based on the rational design, computational design or high-throughput screening have shown increased selectivity for both single- and multi-species biofilms. These methods provide potential therapeutic agents to promote targeted control of the oral microbial biofilms in the near future.

## Introduction

The human oral cavity has over 700 bacterial species, harboring the second most diverse microbiome in the body (Pathak et al., 2021). Dental caries is one of the prominent chronic diseases worldwide. *S. mutans* is considered as the most significant contributor to dental caries due to its cariogenic ability of acid production, acid tolerance and biofilm formation (Pitts et al., 2017; Cui et al., 2019). The biofilm is a structured community that consists of a wide range of microbial species embedded in a self-organized matrix of extracellular polymeric substances (EPSs) (Flemming et al., 2016; Jakubovics et al., 2021). The EPSs not only provide a scaffold for biofilm maturation but also enhance the biofilm tolerance to antimicrobial agents (Jakubovics et al., 2021). The traditional antimicrobial agents, such as the broad-spectrum antimicrobials, pose the challenges for maintaining commensal bacteria (Huang et al., 2021). Though chlorhexidine has a strong bactericidal ability (Coelho et al., 2017), the side effects such as tooth staining, unpleasant taste, mouth irritation, and disturbing the homeostasis of the oral microbiome have limited its usage (Brookes et al., 2020). A larger number of evidence showed that natural products and their derivatives exhibited inhibitory activities against *S. mutans* growth (Sparks et al., 2017). However, the identification and isolation of active compounds from plants are complex and time-consuming. Moreover, the emergence of antibiotic resistance has necessitated the search for novel antibacterial agents target specific oral bacterial pathogens and inhibit EPSs formation (Coelho et al., 2017; Xia et al., 2019).

**Figure 1:**
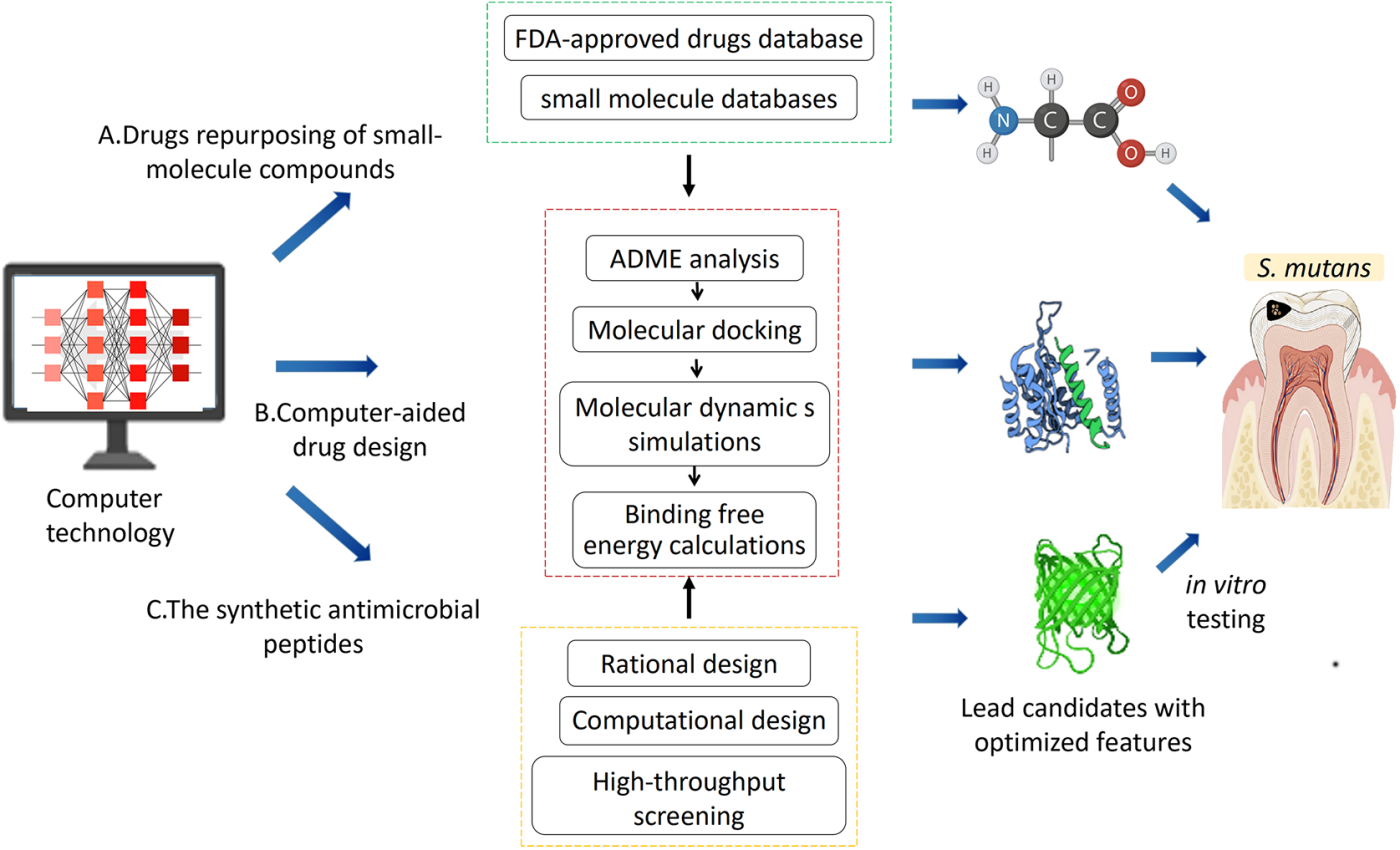
The computer technologies employed to discover novel antimicrobial agents including: **(A)** drug repurposing of small-molecule compounds; **(B)** computer-aided drug design; **(C)** the synthetic antimicrobial peptides. Then *in vitro* testing targeting the *S.mutans* was conducted to select the lead candidates with optimal features.

The ideal anti-biofilm approach is to facilitate the biofilm dispersion, inhibit the reproduction and metabolism of pathogens, as well as avoid the emergence of antibiotic-resistant bacteria and not disturb the homeostasis of the oral microbiome (Kanwar et al., 2017; Simon et al., 2019). Based on FDA-approved drugs database and small molecule database screening, the drug repurposing has advantages of lower toxicity and faster clinical transition (Wishart et al., 2006; Cui et al., 2019). And the rapid advance of computer technology makes it possible to investigate the biomolecular interactions and design/redesign chemical molecules, which enhance a better understanding the mechanism of inhibition and reveal essential structural properties at the molecular level. The antimicrobial peptides (AMPs) have been considered as potential drug candidates (Bin Hafeez et al., 2021), but it is challenging to improve the specificity of AMPs against *S. mutans* (Mai et al., 2011). Computational tools enable exploration of previously unexplored regions of AMP sequence space which may yield synthetic peptides with enhanced biological function (Torres et al., 2021). This review introduced emerging computer approaches including drugs repurposing of small-molecule compounds, computer-aided drug design (CADD), and new synthetic antimicrobial peptides (**Figure 1**).

## Drugs repurposing of small-molecule compounds

Drug repurposing is a method for discovering new uses of the original drugs beyond the scope of the medical indications. Compared to new drug discovery and development, it offers the advantages of lowering drug development cost and risk as existing drugs have already gone through clinical development stages (Ashburn and Thor, 2004). Small molecule compounds, with a molecular weight <1000 Da, have shown good antimicrobial activity, good stability and low toxicity (Xie et al., 2017; Roman, 2021). Recently, a number of comprehensive small molecule databases have emerged including DrugBank (Wishart et al., 2006), ChEMBL (Bento et al., 2014), PharmGKB (Hewett et al., 2002), Zinc (Williams, 2008), and PubChem (Wang et al., 2012), which have improved the chances of success by enabling the pre-selection of active compounds to test *in vitro*. *In silico* prediction of interactions between drugs and target proteins provides a convenient method to predict the new drug–target interactions (DTIs) (Wang and Zeng, 2013). Molecular docking, ligand-based and network-based approaches have been commonly used in virtually screening for a large number of compounds against a target protein (Ekins et al., 2007; Wang and Zeng, 2013). Moreover, Li (Li et al., 2011) have obtained a high enrichment of true positives predictions by using known interaction docking, consensus scoring, and specificity criteria.

Nitro group is critical for the anti–infective activity of nitro-based antimicrobial drugs, including nitroimidazoles, nitrothiazoles, and nitrofurans (Stover et al., 2000; Torreele et al., 2010). Based on the antimicrobial activity of nitrofurans against *S.mutans*, Zhang (Zhang et al., 2019) synthesized a novel water–soluble hybrid of indolin-2-one and nitrofuran ZY354, which exhibited low cytotoxicity and remarkable antimicrobial activity against *S.mutans* in multi-species biofilms. Toremifene, an FDA-approved drug for treating breast cancer, has also shown good inhibitory effect on the growth of *S. mutans* (Gerits et al., 2017a). Kuang (Kuang et al., 2020) identified a natural anticancer compound napabucasin (NAP) showed antimicrobial activity against oral *streptococci*. Using NAP as a lead compound, Xiao (Lyu et al., 2021) redesigned and synthesized a novel molecule LCG-N25. LCG-N25 exhibited stronger antimicrobial activity toward *S.mutans* with lower cytotoxicity.

Vitamin C and vitamin D are essential nutrients to human health. Interestingly, increased evidence showed that salivary vitamin C and serum vitamin D levels were associated with the occurrence of dental caries (Syed et al., 2019). Vitamin C has been reported to inhibit the synthesis of EPSs *via* inhibition of the quorum sensing and other stationary phase regulatory mechanisms (Pandit et al., 2017). Moreover, there was a concentration-dependent inhibitory effect of vitamin C on *S. mutans* growth and biofilm formation (Eydou et al., 2020). Vitamin D plays a key role in tooth mineralization, and it can lead to the “rachitic tooth” if the levels are unregulated (Foster et al., 2014). Saputo (Saputo et al., 2018) had screened FDA-approved drugs to identify old drugs with new therapeutic effects against *S. mutans*, and identified the vitamin D derivative doxercalciferol could interfere with *S. mutans* wall synthesis. Doxercalciferol exhibited synergistic activity in combination with bacitracin and possessed lytic activity against *S. mutans* through a bacitracin resistance mechanism of MbrABCD. Ferumoxytol was an FDA approved nanoparticle to treat iron deficiency (Schwenk, 2010). Liu (Liu et al., 2018) proved that ferumoxytol could disrupt intractable oral biofilms and prevent dental caries *via* intrinsic peroxidase-like activity.

Other drugs, such as dihydrofolate reductase inhibitor, trimetrexate analogues (Zhang et al., 2015), antiasthmatic drug zafirlukast (Gerits et al., 2017b), antifungal azoles lotrimazole and econazole (Qiu et al., 2017), efflux inhibitors reserpine (Zeng et al., 2017) have also been shown as potential inhibitors against *S. mutans* growth (Cui et al., 2019). Dipeptidyl peptidase (DPP IV) is a well-known therapeutic target in Type II diabetes. Anti-human DPP IV drugs saxagliptin can affect *S. mutans* growth (De et al., 2016; De et al., 2018).

Although drug repositioning has advantages such as lower cost, shorter development timelines and higher safety, the side effects and adverse reactions are yet to be solved (Yang et al., 2021). Firstly, the cytotoxicity of the novel molecules, particularly for the synthetic molecules should be comprehensively evaluated before clinical translation. In addition, drug resistance by oral bacteria still requires long-term evaluation both *in vitro* and *in vivo* models (Saputo et al., 2018). And the indications of reused drugs are narrow compared to antibiotics due to their original effects.

## Computer-aided drug design (CADD)

Rapid developments in computer technology makes it possible to investigate biomolecular interactions at the molecular level and design/redesign new chemical molecules through computer-aided drug design (CADD) (Dixon et al., 2006). High research costs and significant decrease in the number of new drug approvals have made commercial pharmaceutical companies hesitant to spend on drug discovery research to some extent (Kitchen et al., 2004). CADD provides information about the bioactive parts of compounds virtually and allow rapid examination of the synthesis processes in a resource-efficient, more reliable, and cost-effective way without actually manufacturing them (Kitchen et al., 2004; Thomford et al., 2018; Xu et al., 2022a). Structure-based virtual screening (SBVS) and ligand-based virtual screening (LBVS) are two main strategies commonly applied in computer-aided drug discovery (Khan et al., 2019). SBVS depends on the structure of the target and interactions with the ligands, while LBVS relies on the central similarity-property principle which indicates that similar molecules should exhibit similar properties (Ekins et al., 2007). The chemical similarity calculations are the core of LBVS (Willett, 2003). The methods of LBVS include similarity and substructure searching, quantitative structure-activity relationships (QSAR), and pharmacophore and 3D shape matching (Lavecchia and Di Giovanni, 2013). On the other hand, SBVS employs the 3D structure of the biological target to dock the candidate molecules and ranks them based on their predicted binding affinity or complementarity to the binding site (Lavecchia and Di Giovanni, 2013). SBVS and LBVS significantly minimize the complexity of finding potential therapeutic compounds against the pathogenic bacterial (Tan et al., 2008). Carmen identified ALS-31 as a small molecule inhibitor of *S. mutans* superoxide dismutase (SOD) by LBVS and SBVS, which inhibited planktonic growth and biofilm formation of *S. mutans* (Cerchia et al., 2022). Pushkaran and his colleagues (Pushkaran et al., 2019) computationally evaluated repurposing of an FDA approved drug Diosmin (DIO) using structure-based drug design method. They identified Diosmin (DIO) targeting the active site residues of L, D-transpeptidase (Ldt) enzymes which involved in *Mycobacterium tuberculosis* (*M. bt*) cell wall biosynthesis.

The process of CADD usually includes the following steps. First, various compounds aimed at the target enzyme undergo high-throughput screening. Then an absorption, distribution, metabolism and excretion (ADME) analysis is performed to determine the pharmacokinetic properties of these screened compounds. Next, molecular docking is applied to investigate the mechanism of interaction between these compounds and the target enzyme structure at the molecular level. Finally, molecular dynamic (MD) simulations and binding free energy calculations are performed to analyze the structure stability (Kitchen et al., 2004; Thomford et al., 2018). Key virulence factors such as antigens I/II, Gtfs and SrtA are usually exploited as the targets for computer-aided drug design against *S. mutans* (**Table 1**).

**Table 1:** Progress in computer-aided drug design (CADD) towards *S.mutans*.

Antimicrobial agents	Chemical Formula	Mechanisms	References
2A4	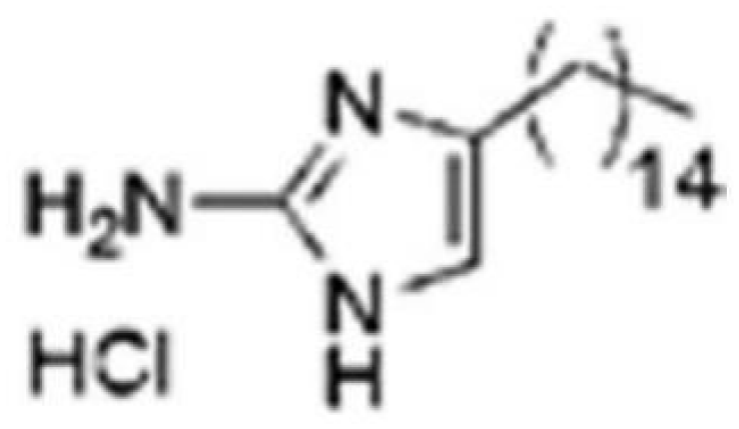	Selectively inhibit *S. mutans* adhesion.	(Liu et al., 2011)
CHEMBL243796	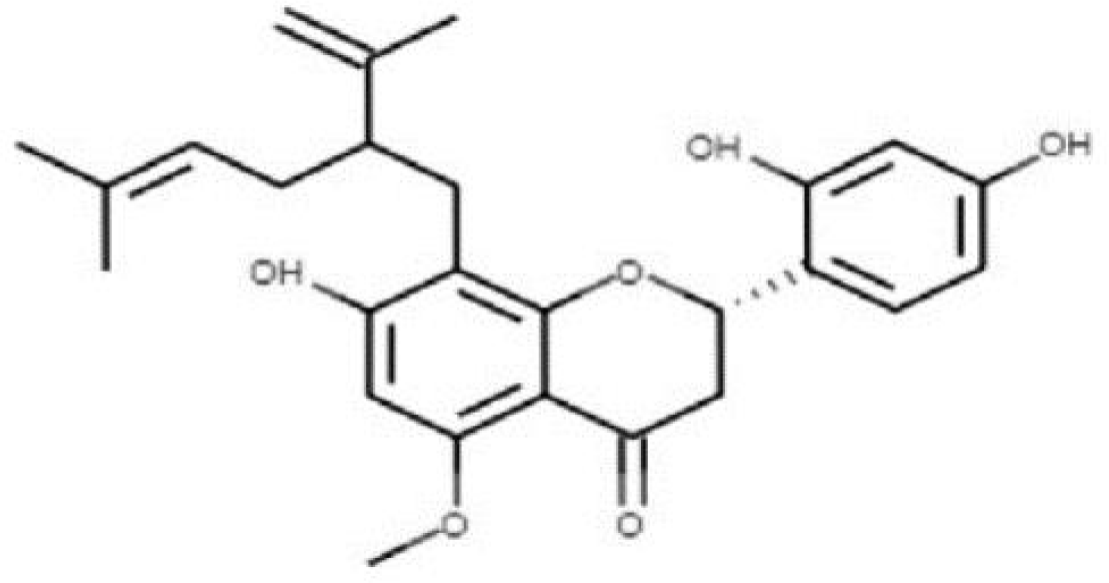	Inhibit *S. mutans* SrtA enzyme activity	(Salmanli et al., 2021)
D25	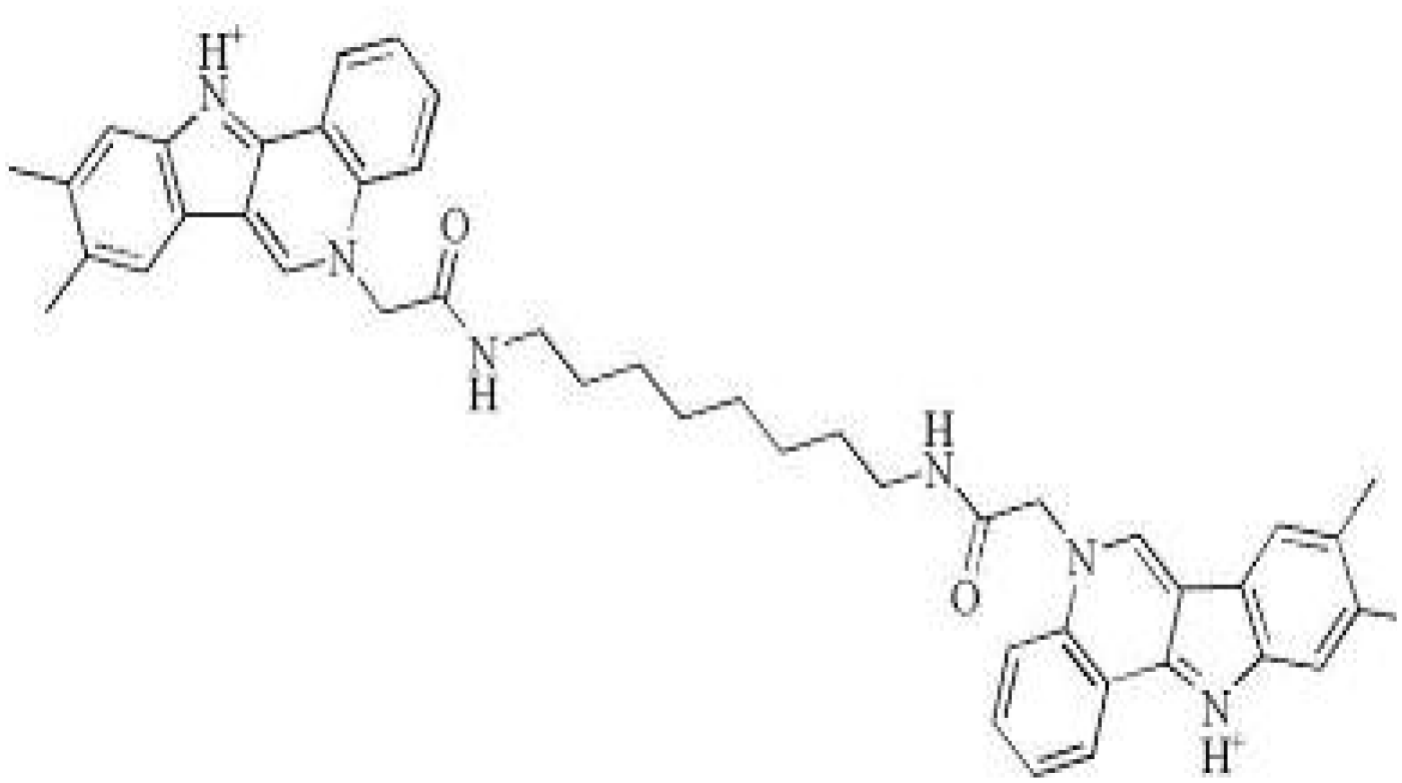	Selectively inhibit antigen I/II and *S. mutans* biofilms	(Chen et al., 2021)
3F1	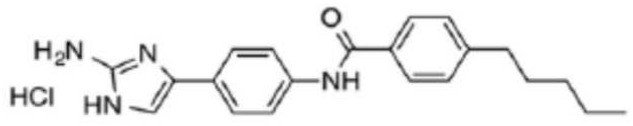	Specifically target *S. mutans* biofilms independently of antigen I/II and Gtfs	(Garcia et al., 2017)
G43	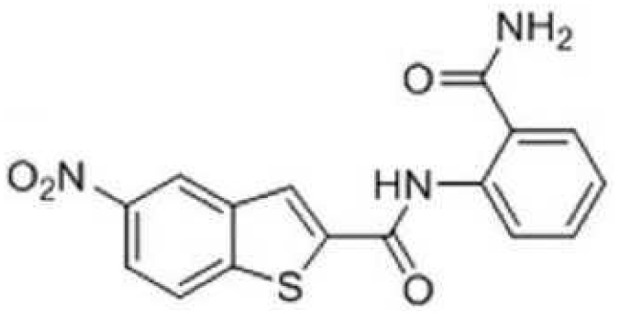	Inhibit *S. mutans* Gtfs and biofilm formation	(Zhang et al., 2017)
2-(4-methoxyphenyl)-N-(3-{[2-(4-methoxyphenyl)ethyl]imino}-1,4-dihydro-2-quinoxalinylidene)ethanamine	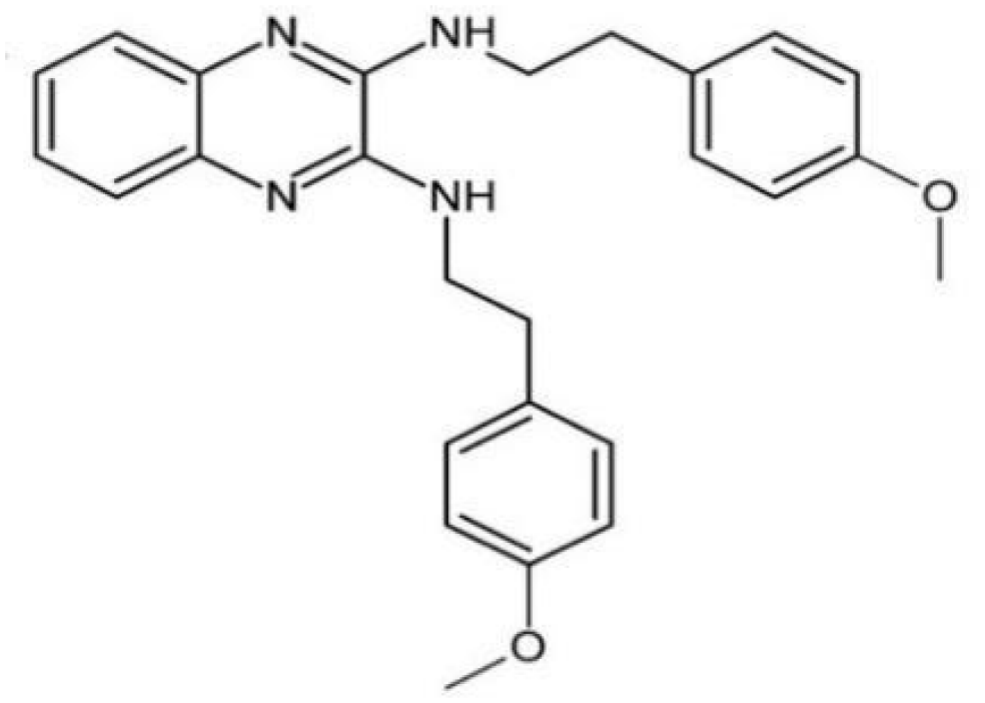	Inhibit EPS synthesis and biofilm formation in *S. mutans* by target GtfC	(Ren et al., 2016)
ZINC19835187 (ZI-187),	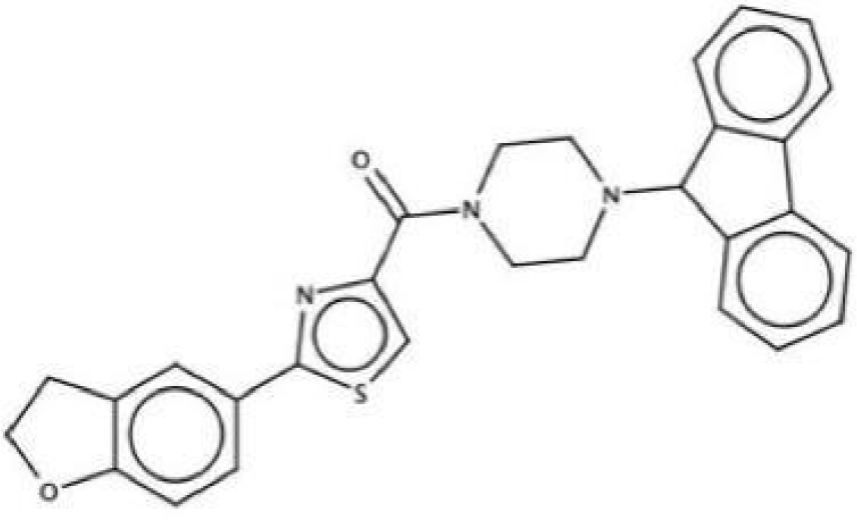	Inhibit *S. mutans* adhesion by binding Ag I/II	(Rivera-Quiroga et al., 2020)
ZINC19924939 (ZI-939)	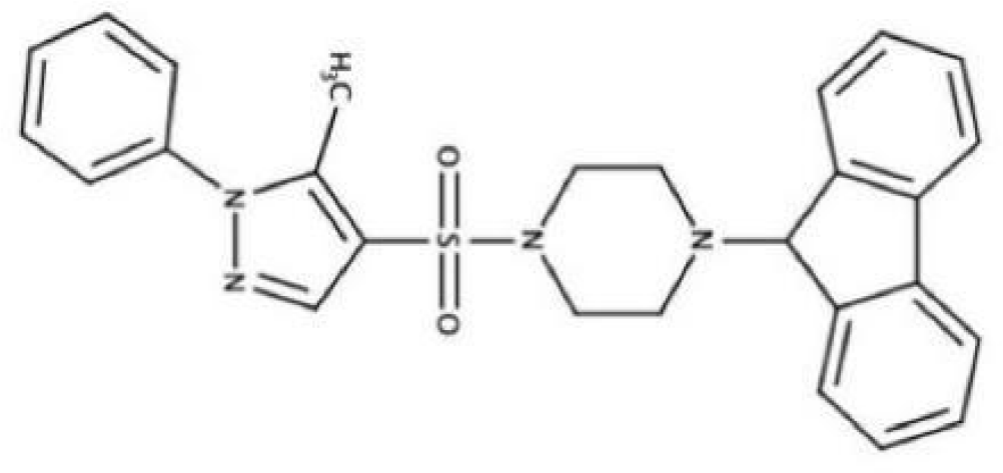		
ZINC19924906 (ZI-906)	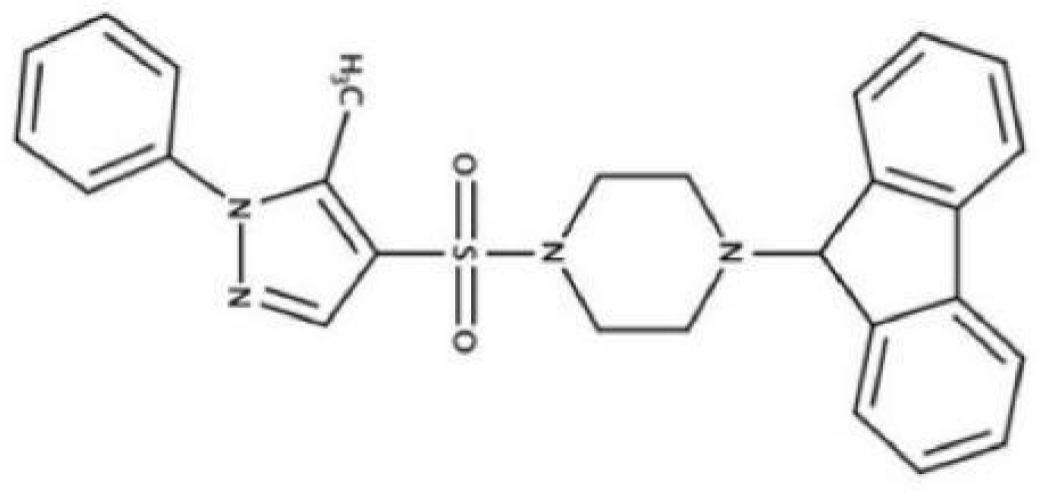		
ZINC95098840	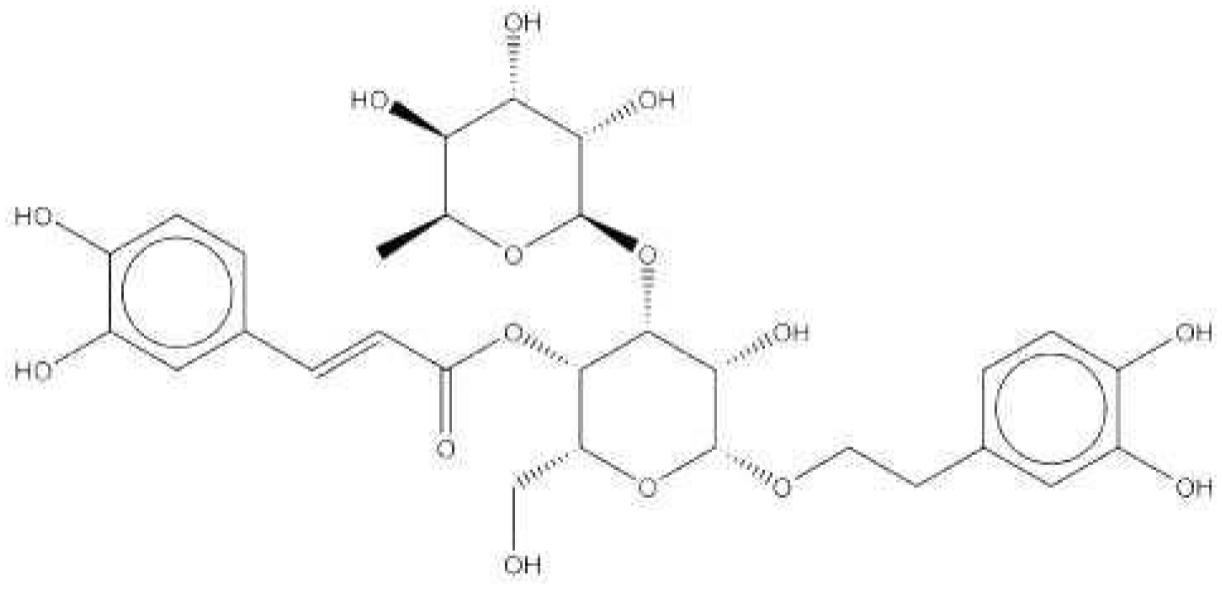	Interact with SrtA and impede SrtA catalysis	(Luo et al., 2017)
ZINC99230413	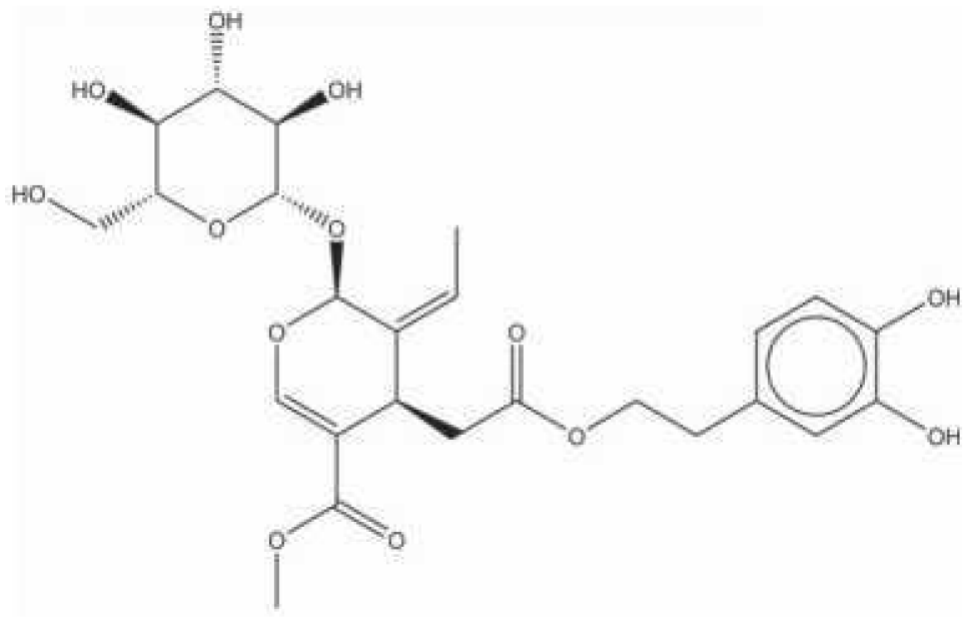		
ZLS-31	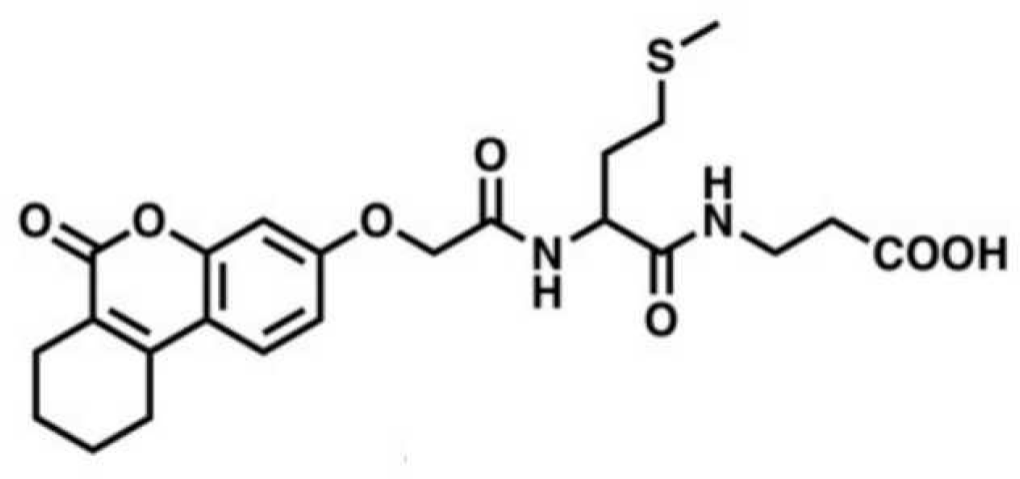	Inhibit *S. mutans* SOD	(Cerchia et al., 2022)

### Antigens I/II

The antigen I/II (Ag I/II) family of adhesins are widely distributed on the cell surface of many streptococci, which also involved in *S. mutans* adhesion to the tooth surface and the bacterial co-aggregation (Matsumoto-Nakano, 2018; Yang et al., 2018). Through virtual searching for inhibitors based on Ag I/II protein structures, Rivera (Rivera-Quiroga et al., 2020) found three molecules ZINC19835187 (ZI-187), ZINC19924939 (ZI-939) and ZINC19924906 (ZI-906) inhibited about 90% adhesion of *S. mutans*. *S. mutans* cell-surface-localized adhesin P1, is an amyloid-forming protein (Tang et al., 2016). The interactions between C123 (C-terminal segment) and P1 contribute to biofilm-related events such as amyloid fibrils formation, suggesting that C3 would serve as a promising anti-amyloid target (Rivière et al., 2020). Chen (Chen et al., 2021) selected small molecules targeting C3 through structure-based virtual screening, and found that D25 selectively inhibited amyloid fibrils and *S. mutans* biofilms but had little influence on biofilms formed by *S. gordonii* and *S. sanguinis*.

### Sortase A

Sortase A (SrtA), one of the membrane-associated sortase enzymes, is responsible for anchoring of numerous virulence-associated surface proteins, including FruA, GbpC, Pac, WapA and Dex, and thus contributes to the biofilm formation of *S. mutans* (Luo et al., 2017). Although small molecules and natural products including trans-chalcone and flavonoid compounds exhibit effective inhibition against SrtA (Hu et al., 2013; Huang et al., 2014; Singh et al., 2014; Panche et al., 2016). The multi-drug resistance and side-effects make the discovery of new inhibitors for SrtA necessary (Luo et al., 2017). After high-throughput screening, CHEMBL243796 (kurarinone) was found to have especially good inhibitory activity against *S. mutans* SrtA (Salmanli et al., 2021). Luo (Luo et al., 2017) revealed that several similar compounds including acteoside (ZINC95098840) and oleuropein (ZINC98230413), with good affinities and appropriate pharmacokinetic parameters, were potential inhibitors to impede the catalysis of SrtA.

### Glucosyltransferases

The glucosyltransferases (Gtfs) of *S. mutans* play essential roles in the etiology and pathogenesis of dental caries. The EPSs, mainly synthesized by Gtfs, provide binding sites that promote accumulation of microorganisms on the tooth surface and further establishment of pathogenic biofilms (Koo et al., 2010). Based on SBVS method, Zhang (Zhang et al., 2017) found two small-molecule compounds #G16 and #G43 specifically inhibited Gtfs and *S. mutans* biofilm formation. The compound #G43 showed great inhibitory effect on the activity of GtfB and GtfC than #G16. Ren (Ren et al., 2016) screened approximately 150,000 compounds from commercially available databases and identified a quinoxaline derivative, 2-(4-methoxyphenyl)-N-(3-{[2-(4-methoxyphenyl)ethyl]imino}-1,4-dihydro-2-quinoxalinylidene) ethanamine as a potential GtfC inhibitor. The computational techniques in drug design have improved the development and optimization of active compounds (Ejalonibu et al., 2021). Liu (Liu et al., 2011) screened a focused small-molecule library and found eight active compounds inhibit *S. mutans* production of Ag I/II and Gtf, shareing similar structural of 2-aminoimidazole (2-AI), which helped to design the derivatives of marine natural products to inhibit both Gram-positive and Gram-negative bacteria.

## The synthetic antimicrobial peptides

Antimicrobial peptides (AMPs) are small bioactive proteins that comprise a part of the body’s first line to inactivate pathogens (Magana et al., 2020). AMPs can inhibit the growth of bacteria, disrupt bacterial cell membrane structure and compete with bacteria for adhesion (Jenssen et al., 2006; Guaní-Guerra et al., 2010; Magana et al., 2020). However, natural antimicrobial peptides generally have limitations, such as a short half-life, unstable in the variable oral environment, and might lead to bacterial resistance (Torres et al., 2019; Niyonsaba et al., 2020). The synthetic AMPs has the advantages including slower emergence of resistance, increased selectivity, and decreased cytotoxicity toward healthy cells (Sullivan et al., 2011; Dalzini et al., 2016; Pletzer et al., 2016). The artificial antimicrobial peptides also have favorable pharmacokinetics and desirable stability (Niu et al., 2021a; Niu et al., 2021b). The synthesized antimicrobial peptide SET-M33D, an isomeric form with D amino acids, can kill multi-resistant pathogens, including Gram-positive *S. aureus*, *S. saprophyticus*, and various Gram-negative *Enterobacteriaceae* with high efficacy and low toxicity (Brunetti et al., 2020). The synthesized antimicrobial peptide GH12 induced low toxicity in human gingival fibroblasts and significantly reduce the cariogenic properties of *S. mutans* by decreasing the lactic acid production and water-insoluble EPS synthesis (Wang et al., 2018). GH12 could make *S. sanguinis* and *S. gordonii* expand their ecological advantages by promoting hydrogen peroxide production, shifting the microbial composition to a more balanced one (Jiang et al., 2018; Jiang et al., 2020).

The methods of AMPs synthesis or discovery can be grouped into three approaches: rational design, computational design and high-throughput screening (Torres et al., 2019; Lei et al., 2021).

### Rational design

The fusion of targeting and killing peptides is a common rational design strategy (Guo et al., 2015). A new class of pathogen-selective molecules, called selectively or specifically targeted antimicrobial peptides (STAMPs) was constructed (Eckert et al., 2006b; Huo et al., 2018). STAMP contains a pathogen-specific target peptide and antimicrobial peptide and/or connecting regions can selectively kill bacteria (Huo et al., 2018). *S. mutans* quorum-sensing (QS) system regulates the gene expression, bacteriocin production and biological behavior partially through competence stimulating peptide (CSP) (Huo et al., 2018). CSP serves as a STAMP targeting domain to mediate *S. mutans*-specific delivery of the antimicrobial peptide domain (Eckert et al., 2006a). When fused with broad antimicrobial peptide G2 at either the C terminus or N terminus, the CSP-derived STAMP C16G2, M8G2 and C16-33 showed robust, specific activity against *S. mutans* grown in planktonic cultures and biofilms in both single and multi-species biofilm states (Eckert et al., 2006a; Sullivan et al., 2011; De La Fuente-Nunez, 2019). The STAMPs C16G2, M8G2, C16-33, and M8-33 can target *S. mutans* without disturbing noncariogenic oral streptococci, indicating that they can maintain a normal ecological balance (Tan et al., 2008). Li (Li et al., 2010) synthesized *S. mutans*-specific targeting peptide 2_1G2, a derivative in which the *S. mutans*-specific CSPC16 targeting domain was replaced with peptide 2_1. The STAMP 2_1G2 could lead to protective biofilms formation with the ability to prevent secondary surface colonization by cariogenic *S. mutans*. A series of STAMPs C8H, C11H, C12H, C13H, and C14H were synthesized, and their selective antibacterial activity against *S. mutans* on single species and multi-species biofilms were studied, and a total of 21 protein spots were downregulated after C11H treatment.

### Computational design

Advances in computational-resources have facilitated the discovery and synthesis of novel AMPs (Porto et al., 2017; De La Fuente-Nunez, 2019), including structure-activity relationships (SAR) study (Abdel Monaim et al., 2018), neural networks (Müller et al., 2018), deep learning (Hamid and Friedberg, 2019). Since understanding the role and importance of each amino acid residue in a given sequence is fundamental for programming peptide, quantitative structure–activity relationship studies (QSAR) have been used to describe amino acid residues and their features (Torres et al., 2021). The approaches applied for the identification of AMPs from databases, including local alignments, regular expressions (REGEX), activity prediction by machine learning (Porto et al., 2017). By using supervised machine learning and a genetic algorithm, Boone (Boone et al., 2021) found a peptide active against *S. epidermidis*, with an improved ease of synthesis. Yazici (Yazici et al., 2016) analyzed the structures and predicted ternary conformations of the engineered chimeric peptides through computational modeling methods, which exhibited antimicrobial activity against *S. mutans*, *S. epidermidis*, and *E. coli*.

### High-throughput screening

High-throughput screening of peptides is also an effective way. The main advantage of large screens is the higher probability of obtaining hits, which helping to select and optimize complex molecular descriptors, and obtain a more precise definition of simple molecular descriptors (Torres et al., 2021). Xie have developed an effective high-throughput screening system for designing and screening peptides that acted selectively on microbial membranes (Xie et al., 2006). However, the reported number of molecules needed to achieve conclusive SAR studies is higher than that required for structure-based screening approaches (Torres et al., 2021).

## Conclusion and future prospects

Traditional anti-caries agents, such as fluoride, chlorhexidine, and antibiotics, are characterized by side-effects and low selectivity, which may destroy the homeostasis of oral microbiome. More attention is being directed to find alternative agents to control biofilm-related diseases. Drug repurposing of small molecule compounds lower drug development cost and risk. However, it has come to a consensus that preventing or treating dental biofilms are particularly challenging with small-molecule drugs due to low solubility, brief topical-exposure regimens, salivary clearance, and limited drug diffusion into EPS biofilm matrix (Sims et al., 2020; Osman et al., 2022). Nanoparticle carriers have shown good ability to increase small- molecule drug penetration to biofilms, improve drug stability, and enhance drug bioavailability (Sims et al., 2020; Zhang et al., 2021), which may be potential candidates for clinical translation (Xu et al., 2022b). CADD provides information about the bioactive parts of compounds virtually and allow rapid examination of the synthesis processes. Using friendly and publicly accessible web-servers may be the future direction of prediction methods and computational tools development (Porto et al., 2018). And one of the limitations of computer-guided methods is the need for standardized and reliable biological data as input for designing process. The new synthesized antimicrobial peptide showed increased selectivity and decreased cytotoxicity, but AMPs perform poorly in the oral cavity due to low target specificity in solution, anionic protein adsorption and the diluting effects of saliva (Ramburrun et al., 2021). Designing hydroxyapatite-binding antimicrobial peptide (HBAMP), or incorporation into liquid crystalline systems (LCS) may be effective in the delivery of peptides, more strategies are still required to improve AMPs physiological *in vitro* and *in vivo* stability.

Moreover, it is noteworthy that current studies have mainly focused on *in vitro* or animal studies using single-species biofilms, which would limit the clinical translation of these approaches. The mechanism of small-molecule compounds’ inhibitory effect on the biofilm is still unclear. The multi-species microbial, saliva, and enzyme make the oral cavity environment complex, affecting the efficacy of the novel agents *in vivo*. Combining these new methods, such as computational tools and algorithms, may be an effective way to synthesize novel antimicrobial agents. Further studies are also necessary to evaluate the antimicrobial activities, bioactivity, and biocompatibility of the novel drug more comprehensively and find effective drug delivery systems.

## Author contributions

BZ contributed to original draft preparation and writing. MZ and JT contributed to review and editing. LL and RH contributed to revise the manuscript. All authors contributed to writing and reviewing the manuscript.

## Funding

This work was funded by the Health Research Fund of Shaanxi Province (NO. 2021E020) and Interdisciplinary Innovation Projects of West China Hospital of Stomatology (RD-03-202103).

## Conflict of interest

The authors declare that the research was conducted in the absence of any commercial or financial relationships that could be construed as a potential conflict of interest.

## Publisher’s note

All claims expressed in this article are solely those of the authors and do not necessarily represent those of their affiliated organizations, or those of the publisher, the editors and the reviewers. Any product that may be evaluated in this article, or claim that may be made by its manufacturer, is not guaranteed or endorsed by the publisher.
